# Juvenile Hemochromatosis in an Asymptomatic Patient—Importance of Early Diagnosis

**DOI:** 10.1097/PG9.0000000000000228

**Published:** 2022-07-29

**Authors:** Bola Nashed, Carolina Fonseca, William Vander Pols, Sanjay Kumar, Hernando Lyons, Brian Berman, Gianna M. Guzzardo

**Affiliations:** From the * Department of Internal Medicine, Ascension St. John Hospital, Detroit, MI; † Department of Pediatrics, Ascension St. John Children’s Hospital, Detroit, MI; ‡ Michigan State University College of Human Medicine, East Lansing, MI; § Department of Pediatric Gastroenterology, Ascension St. John Children’s Hospital, Detroit, MI; ∥ Division of Pediatric Hematology Oncology, Children’s Hospital of Michigan, Detroit, MI; ¶ Central Michigan University College of Medicine, Detroit, MI.

**Keywords:** juvenile hemochromatosis, elevated transaminases, phlebotomy, cirrhosis, iron overload

## Abstract

Juvenile hemochromatosis is a rare inherited disorder of iron regulation leading to iron overload, which usually presents before the age of 30. One of the most serious clinical characteristics associated with early-onset iron overload is liver disease with eventual cirrhosis, often associated with a reduced life expectancy even after treatment. This case report summarizes an asymptomatic pediatric patient with persistently elevated transaminase levels, which led to a diagnosis of juvenile hemochromatosis relatively early in the course of his disease. The aim of this case report is to increase awareness and stress the importance of early diagnosis and treatment, as it is vital to prevent life-threatening complications and optimize patient outcomes. Consideration should be taken to recognize potential manifestations despite the rarity of the condition. Patients with signs of hepatocellular injury without explanation should prompt evaluation including consideration for iron overload after other common causes are ruled out.

## INTRODUCTION

Juvenile hemochromatosis (JH), also known as hereditary hemochromatosis type 2, is a rare and severe autosomal recessive disease that presents with iron overload and has an aggressive clinical course (1). Hemojuvelin (HJV) gene encodes hemojuvelin, which regulates hepcidin production. Its mutations account for over 90% of cases of JH; the variant p.Gly320Val is the most prevalent pathogenic variant reported to date (2). Hepcidin blocks the release of iron from enterocytes and macrophages into the circulation (1). The absence of HJV activity leads to defective hepcidin production resulting in circulatory iron overload. This results in deposition of excess iron in tissues leading to organ damage (3).

One of the more serious sequela of JH is liver disease with eventual cirrhosis, which has been associated with reduced life expectancy unless treated early. Other potential severe complications include cardiomyopathy, hypogonadotropic hypogonadism, diabetes, and arthropathy (3).

## CASE

A 9-year-old male presented to our pediatric gastroenterology clinic for evaluation of elevated transaminase levels (AST61IU/L, ALT110IU/L) that were found incidentally as part of evaluation for cervical lymphadenopathy. Laboratory tests were obtained before any treatment, and lymphadenopathy resolved after a short course of antibiotics. He had a history of asthma and was on montelukast and budesonide without additional comorbidities. Physical examination revealed a thin child with a BMI in the 18th percentile but was otherwise unremarkable. Laboratory studies for infectious etiologies, Wilson’s disease, alpha-1-antitrypsin deficiency, fatty liver disease and autoimmune hepatitis were normal. Repeat liver enzymes four months later showed persistent elevation in transaminase levels despite a normal liver ultrasound. To rule out possible drug-induced liver injury, montelukast was discontinued, but transaminase levels remained elevated (AST 91 IU/L; ALT 121 IU/L). All other tests including alkaline phosphatase, gamma-glutamyl transferase, total bilirubin, and albumin were normal. An iron panel revealed elevated ferritin level (1367 ng/mL), serum iron (273 µg/dL), TIBC (290 µg/dL), iron saturation (94.1%), and serum transferrin (229 mg/dL). These results implied circulatory iron overload, so a genetic panel for hemochromatosis was obtained that showed double heterozygosity for two pathogenic mutations in the HJV gene (p.Leu366* on exon 4 and p.Asp149Thrfs*97 on exon 3), confirming a diagnosis of JH. Both parents were confirmed carriers for each HJV variant mutation.

Liver MRI demonstrated mild hepatomegaly (span of 15 cm), diffusely low T2 signal, and liver iron concentration between 14.46 and 19.13 mg iron/gm of dry liver (normal range 0.2–2 mg iron/gm of dry liver). Liver fibroscan showed grade F0-1 fibrosis and grade S1 steatosis. In the absence of clinical and laboratory signs of cirrhosis, and significant evidence of fibrosis on liver elastography, we elected not to perform the liver biopsy.

**FIGURE 1.**
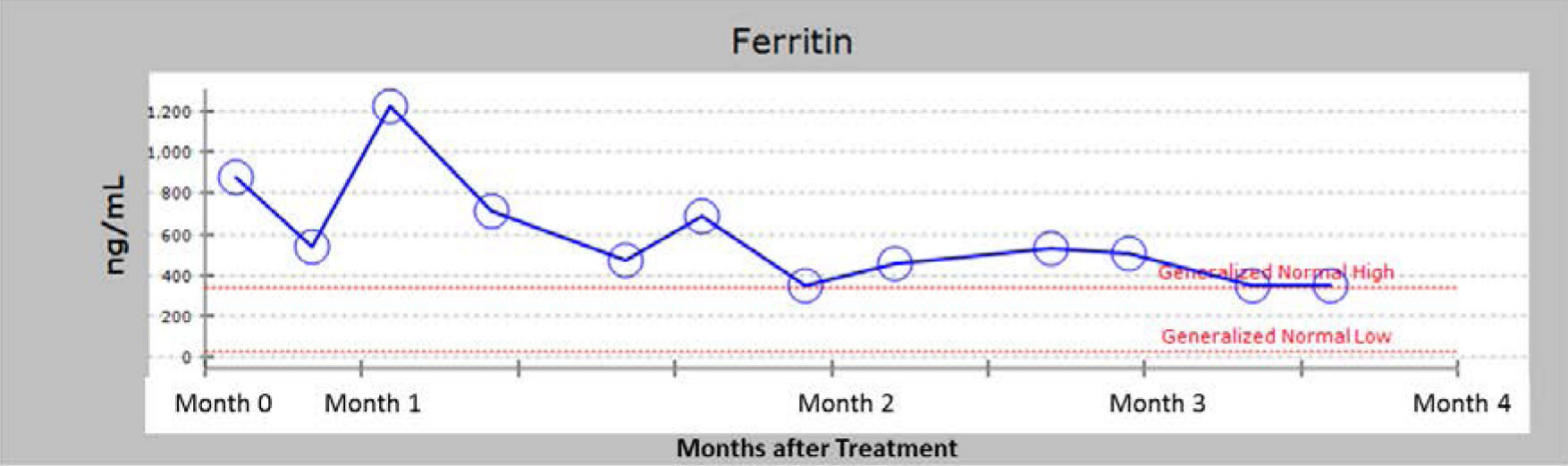
Patient’s ferritin levels while receiving weekly phlebotomy therapy.

EKG and echocardiogram were normal. Cardiac MRI demonstrated normal structure and function. The cardiac T2 value was calculated at 40–49 ms (normal range >20 ms), reflecting no significant cardiac iron overload. Brain MRI revealed T1 hyper-intensity involving bilateral globus pallidus, internal capsule, ventral thalamus, and also showed intrinsic T1 hyper-intensity of the ventral aspect of the anterior pituitary gland correlating with iron overload. He was referred to a pediatric endocrinologist who found normal endocrine function. Hematology instituted weekly phlebotomy therapy. Since starting treatments, he has had a gradual decline inferritin levels (Fig. 1), and after 6 months, he had marked improvement in iron deposition (liver iron concentration decreased to 5.3 mg iron/gm of dry weight liver). He continues on less aggressive phlebotomy treatment, as his serum ferritin level is approaching the normal range.

## DISCUSSION

Because the presentation of JH varies with the severity of iron overload, it can be easily overlooked. Nonspecific symptoms such as fatigue and decreased appetite generally precede the development of organ dysfunction. Liver involvement can manifest as asymptomatic elevation of liver enzymes, hepatomegaly, liver fibrosis (44%–58% of cases), cirrhosis (27%–42% of cases), and rarely hepatocellular carcinoma. Iron deposition in the anterior pituitary gland may lead to hypogonadotropic hypogonadism (67%–91% of cases), and diabetes may be seen (30%–57% of cases) because of pancreatic fibrosis. Cardiac disease, including arrhythmias and cardiomyopathy (35%–37% of cases), is the most common cause of death. Iron deposition in joints may lead to arthralgia usually affecting metacarpophalangeal joints. Increase in skin pigmentation is seen in 24% of cases (2).

A ferritin level above the normal range for age and sex and transferrin saturation >45% in a patient without acute inflammation is suggestive of hemochromatosis. Diagnosis is confirmed through genetic testing (2). Following confirmation, the extent of other organ involvement should be established (4).

Phlebotomy is the preferred treatment. Iron chelators including deferasirox may be used if phlebotomy is contraindicated. Patients with severe iron overload may require both treatments. Phlebotomy can be performed weekly in the induction phase with a ferritin level target of approximately 50 μg/L (5), after which phlebotomy is done less frequently to maintain normal ferritin and transferrin saturation levels (6). Early phlebotomy therapy can halt the progression of organ injury, but depending on severity may not completely reverse organ dysfunction (2).

Diagnosis and management of JH is vital to optimize patient outcomes. We recommend obtaining ferritin and transferrin levels for patients with signs of hepatocellular injury without clear explanation. Clinicians should also recognize unexplained endocrine or cardiac dysfunction as potential early indicators of JH. Genetic testing should be performed to confirm the diagnosis, and MRI studies are important to assess the extent of iron deposition. Phlebotomy is the primary therapeutic approach. Although JH is extremely rare, clinicians should keep it on their differential because early treatment may prevent complications and end-organ damage.

## ACKNOWLEDGMENTS

Dr Nashed (article guarantor) participated in creating the draft and reviewing the literature. Dr Fonseca and Dr Vander Pols, participated in creating the draft and reviewing the literature. Dr Kumar, Dr Lyons, Dr Berman, and Dr Guzzardo participated in revising the draft and approving it for submission.
